# Sequential Mixed Cultures: From Syngas to Malic Acid

**DOI:** 10.3389/fmicb.2016.00891

**Published:** 2016-06-21

**Authors:** Florian Oswald, Stefan Dörsam, Nicolas Veith, Michaela Zwick, Anke Neumann, Katrin Ochsenreither, Christoph Syldatk

**Affiliations:** Technical Biology, Institute of Process Engineering in Life Sciences, Karlsruhe Institute of TechnologyKarlsruhe, Germany

**Keywords:** syngas, fermentation, *Clostridium ljungdahlii*, *Aspergillus oryzae*, malic acid, acetate, process coupling

## Abstract

Synthesis gas (syngas) fermentation using acetogenic bacteria is an approach for production of bulk chemicals like acetate, ethanol, butanol, or 2,3-butandiol avoiding the fuel vs. food debate by using carbon monoxide, carbon dioxide, and hydrogen from gasification of biomass or industrial waste gases. Suffering from energetic limitations, yields of C_4_-molecules produced by syngas fermentation are quite low compared with ABE fermentation using sugars as a substrate. On the other hand, fungal production of malic acid has high yields of product per gram metabolized substrate but is currently limited to sugar containing substrates. In this study, it was possible to show that *Aspergilus oryzae* is able to produce malic acid using acetate as sole carbon source which is a main product of acetogenic syngas fermentation. Bioreactor cultivations were conducted in 2.5 L stirred tank reactors. During the syngas fermentation part of the sequential mixed culture, *Clostridium ljungdahlii* was grown in modified Tanner medium and sparged with 20 mL/min of artificial syngas mimicking a composition of clean syngas from entrained bed gasification of straw (32.5 vol-% CO, 32.5 vol-% H_2_, 16 vol-% CO_2_, and 19 vol-% N_2_) using a microsparger. Syngas consumption was monitored via automated gas chromatographic measurement of the off-gas. For the fungal fermentation part gas sparging was switched to 0.6 L/min of air and a standard sparger. Ammonia content of medium for syngas fermentation was reduced to 0.33 g/L NH_4_Cl to meet the requirements for fungal production of dicarboxylic acids. Malic acid production performance of *A. oryzae* in organic acid production medium and syngas medium with acetate as sole carbon source was verified and gave Y_P∕S_ values of 0.28 g/g and 0.37 g/g respectively. Growth and acetate formation of *C. ljungdahlii* during syngas fermentation were not affected by the reduced ammonia content and 66 % of the consumed syngas was converted to acetate. The overall conversion of CO and H_2_ into malic acid was calculated to be 3.5 g malic acid per mol of consumed syngas or 0.22 g malic acid per gram of syngas.

## Introduction

Nowadays most bulk chemicals are still based on fossil fuels like crude oil and natural gas. It is consensus that, due to dwindling resources and climate change, it is necessary to develop sustainable methods for the production of industrially relevant chemicals. Suitable candidates for these demands are various dicarboxylic acids because of their suitability to be used for the synthesis of various polymers, as was summarized by Lee et al. (2011). In 2004, the US Department of Energy selected the C_4_ dicarboxylic acids malic acid, fumaric acid and succinic acid to be one of the 12 most important platform chemicals produced from biomass (Werpy and Petersen, 2004). However, malic acid is still mostly produced from petroleum (Lohbeck et al., 2000, Miltenberger, 2000). It can be used for the synthesis of polymers for the food and pharmaceutical industries (Werpy and Petersen, 2004), as well as for many other bulk and fine chemicals. Some fungi from the genus *Aspergillus*, like *Aspergilus flavus* or *Aspergilus oryzae*, produce, under certain stress conditions, sizeable amounts of malic acid which is secreted to the culture medium. The development and optimization of production processes for *A. flavus* in the last decades leads to possible malic acid yields of 0.94 g/g using glucose as carbon source (Peleg et al., 1988, 1989; Battat et al., 1991). A major disadvantage of *A. flavus* is the production of aflatoxins which makes its usage in fields of medicine or food industry impossible. The close relative, but not aflatoxin producing *A. oryzae* has also been investigated for the production of malic acid (Knuf et al., 2013). A strain developed by metabolic engineering achieved concentrations of 154 g/L from 160 g/L glucose (Brown et al., 2013). It was also possible to establish processes for malic acid production with metabolically engineered model organisms like *E. coli* or *S. cerevisiae* based on glucose (Moon et al., 2008; Zelle et al., 2008; Zhang et al., 2011). It could be shown that *A. oryzae* is able to convert several alternative carbon sources to malic acid, like glycerol or pentose sugars (i.e., xylose), which are also part of lignocellulosic material (Ochsenreither et al., 2014). The “food or fuel” debate shows the importance of developing biotechnological malic acid production based on sustainable carbon sources which are not competing with food or feed production. Therefore, alternative carbon sources based on lignocellulose, such as hydrolysates from lignocellulose separation or pyrolysis oil from thermal treatment of lignocellulosic biomass as substrate for malate production by *Aspergilli* would be conceivable. Other attempts to produce C_4_-molecules from lignocellulosic substrates focus on the production of n-butanol or butyric acid using anaerobic bacteria (Zhang et al., 2009). The most popular process for butanol production from anaerobic microorganisms is ABE (Acetone, Butanol, Ethanol) fermentation, which uses solventogenic bacteria of the genus *Clostridium*. Using different lignocellulosic and hemicellulosic substrates, final butanol concentrations of up to 18 g/L are reported (Schiel-Bengelsdorf et al., 2013). As for other biotechnological processes, ABE fermentation is limited to sugar containing substrates. In recent years industrial exhaust gases like steel mill off-gas (Köpke et al., 2011) and syngas, a mixture of H_2_, CO, and CO_2_, from gasification of biomass and waste streams like sewage sludge and municipal waste (Hammerschmidt et al., 2011, Rokni, 2015) as well as other C_1_ molecules came into focus as interesting substrates for biotechnological applications (Daniell et al., 2012; Bengelsdorf et al., 2013). Bacteria able to grow on hydrogen and carbon monoxide/carbon dioxide as sole energy and carbon source are called acetogens (Diekert and Wohlfarth, 1994). They use a metabolic pathway unique to this class of bacteria (Schuchmann and Müller, 2014) which incorporates two molecules of CO or CO_2_ via subsequent reactions into one molecule of acetyl-CoA (Diekert and Wohlfarth, 1994). This pathway is known as Wood-Ljungdahl-pathway or acetyl-CoA-pathway (Schuchmann and Müller, 2014). Further conversion of acetyl-CoA yields acetate, ethanol, butyrate, butanol, or 2,3-butandiol as natural products of this pathway. Of these, the formation of C_2_-molecules (acetate and ethanol) has the highest energy gain for acetogenic bacteria (Bengelsdorf et al., 2013). Therefore, C_2_-molecules are the preferred products with reported concentrations of up to 44 g/L (Demler and Weuster-Botz, 2011). Reported concentrations for butyrate, butanol and 2,3-butanediol are low compared to acetic acid (Neumann et al., 2015). More details about the metabolism of acetogenic bacteria can be found in Schuchmann and Müller (2014) or Bengelsdorf et al. (2013).

Neither are acetogenic bacteria able to produce dicarboxylic acids like malic acid or fumaric acid, as their product spectrum is limited due to energetic reasons, nor are *Aspergilli* able to use C_1_-molecules like CO or CO_2_ as fermentation substrates. Therefore, this study focuses on broadening the substrate spectrum for production of malic acid or any other biotechnological product beyond glycerol and sugars and simultaneously expanding the product spectrum of syngas fermentation. By sequential coupling of anaerobic syngas fermentation and aerobic malic acid production the substrate spectrum is broadened to syngas, which can be obtained from steel mill off-gas (Köpke et al., 2011) or by gasification of biomass organic wastes and fossil feedstocks (Neumann et al., 2015), forging a completely new and highly innovative path toward the establishment of a biobased economy.

## Materials and methods

### Microorganisms and medium

If not stated differently all chemicals were purchased from Carl-Roth (Germany) or Sigma-Aldrich (Germany).The organism used for the syngas fermentation part of the study was *Clostridium ljungdahlii* DSM13528 which was kindly provided by the group of Peter Dürre, University of Ulm. Medium used for cultivation of *C. ljungdahlii* for both flask and bioreactor cultivation was based on Tanner (2007). Medium for maintenance and pre-culture cultivation contained: 20 g/L 2-(*N*-morpholino) ethansulfonic acid (MES), 0.5 g/L yeast extract (BD, USA), 2 g/L NaCl, 2.5 g/L NH_4_Cl, 0.25 g/L KCl, 0.25 g/L KH_2_PO_4_, 0.5 g/L MgSO_4_·7 H_2_O, 0.1 g/L CaCl_2_·2 H_2_O, 10 mL trace element solution (composition see below), 10 mL vitamin solution (composition see below) and 0.001 g/L resazurin and was prepared using strict anaerobic techniques. The pH was adjusted to 5.9 using KOH before bottling. Bottles were anaerobized using a gas mixture containing 20 vol-% carbon dioxide in nitrogen (Air Liquide, France). After autoclaving at 121 °C, 1 g Cysteine-HCl·H_2_O and 10 g fructose per liter were added. Trace element solution contained: 2 g/L nitrilotriacetic acid, 1 g/L MnSO_4_·H_2_O, 0.567 g/L FeSO_4_·7 H_2_O, 0.2 g/L CoCl_2_·6 H_2_O (Riedel-de Haën, Germany), 0.2 g/L ZnSO_4_·7 H_2_O, 0.02 g/L CuCl_2_·2 H_2_O, 0.02 g/L NiCl_2_·6 H_2_O, 0.02 g/L Na_2_MoO_4_·2 H_2_O, 0.02 g/L Na_2_SeO_3_·5 H_2_O, and 0.022 g/L Na_2_WO_4_·2 H_2_O. Vitamin solution used for all anaerobic medium in this work contained: 0.002 g/L biotin, 0.002 g/L folic acid, 0.01 g/L pyridoxine (Alfa Aesar, Germany), 0.005 g/L thiamine-HCl, 0.005 g/L riboflavin, 0.005 g/L niacin, 0.005 g/L Ca-pantothenate, 0.005 g/L cobalamin, 0.005 g/L 4-aminobenzoic acid, and 0.005 g/L liponic acid (Cayman Chemical, USA). Maintenance cultures were cultivated at 37 °C without shaking and inoculated every 4 days using the latest maintenance culture.

The *A. oryzae* DSM1863 strain was received from DSMZ strain collection (Deutsche Sammlung von Mikroorganismen und Zellkulturen, Braunschweig, Germany) and was grown on minimal medium (MM) for *Aspergillus* species (Barratt et al., 1965): 6 g/L NaNO_3_, 0.52 g/L KCl, 0.52 g/L MgSO_4_· 7 H_2_O, and1.52 g/L KH_2_PO_4_. The pH was set to 6.5 with 4M NaOH. 2 mL of 1000 × Hutner's Trace Elements, 10 g/L glucose, and 15 g/L agar were added, and the medium was sterilized by autoclaving. 1000 × Hutner's Trace Element solution contained 5 g/L FeSO_4_·7 H_2_O, 50 g/L EDTA-Na_2_, 22 g/L ZnSO_4_·7 H_2_O, 11 g/L H_3_BO_3_, 5 g/L MnCl_2_·4 H_2_O, 1.6 g/L CoCl_2_·6 H_2_O, 1.6 g/L CuSO_4_·5 H_2_O, and 1.1 g/L (NH_4_)_6_Mo_7_O_24_·4 H_2_O, pH 6.5 (Barratt et al., 1965). For conidia collection, the fungus was grown on high-salt minimal medium (Song et al., 2001) which additionally contained 22.37 g/L KCl. Conidia were harvested with 50 % glycerol solution from plates that were incubated for 5 days at 30 °C and filtered through Miracloth (Calbiochem). The conidia solution was diluted to a concentration of 10^7^ conidia/mL and stored at −80 °C. Sequential malic acid production was accomplished in a two-step process with a pre-culture and a main culture. The main culture was either the fermentation broth from *C. ljungdahlii* syngas-fermentation (see above) or main culture medium for fungal malic acid production (Ochsenreither et al., 2014). The pre-culture medium contained 40 g/L glucose monohydrate, 4 g/L (NH_4_)_2_SO_4_, 0.75 g/L KH_2_PO_4_, 0.98 g/L K_2_HPO_4_·3H_2_O, 0.1 g/L MgSO_4_·7 H_2_O, 0.1 g/L CaCl_2_·2 H_2_O, 5 mg/L NaCl, and 5 mg/L FeSO_4_·7 H_2_O and was sterilized by autoclaving for 20 min at 121 °C. Main culture medium contained 120 g/L glucose monohydrate, 1.2 g/L (NH_4_)_2_SO_4_, 0.1 g/L KH_2_PO_4_, 0.17 g/L K_2_HPO_4_·3H_2_O, 0.1 g/L MgSO_4_·7 H_2_O, 0.1 g/L CaCl_2_·2 H_2_O, 5 mg/L NaCl, and 60 mg/L FeSO_4_·7 H_2_O. To keep the pH above 5.5 during acid production 90 g per liter CaCO_3_ were added.

### Fermentation setup and operation

**Figure 1:**
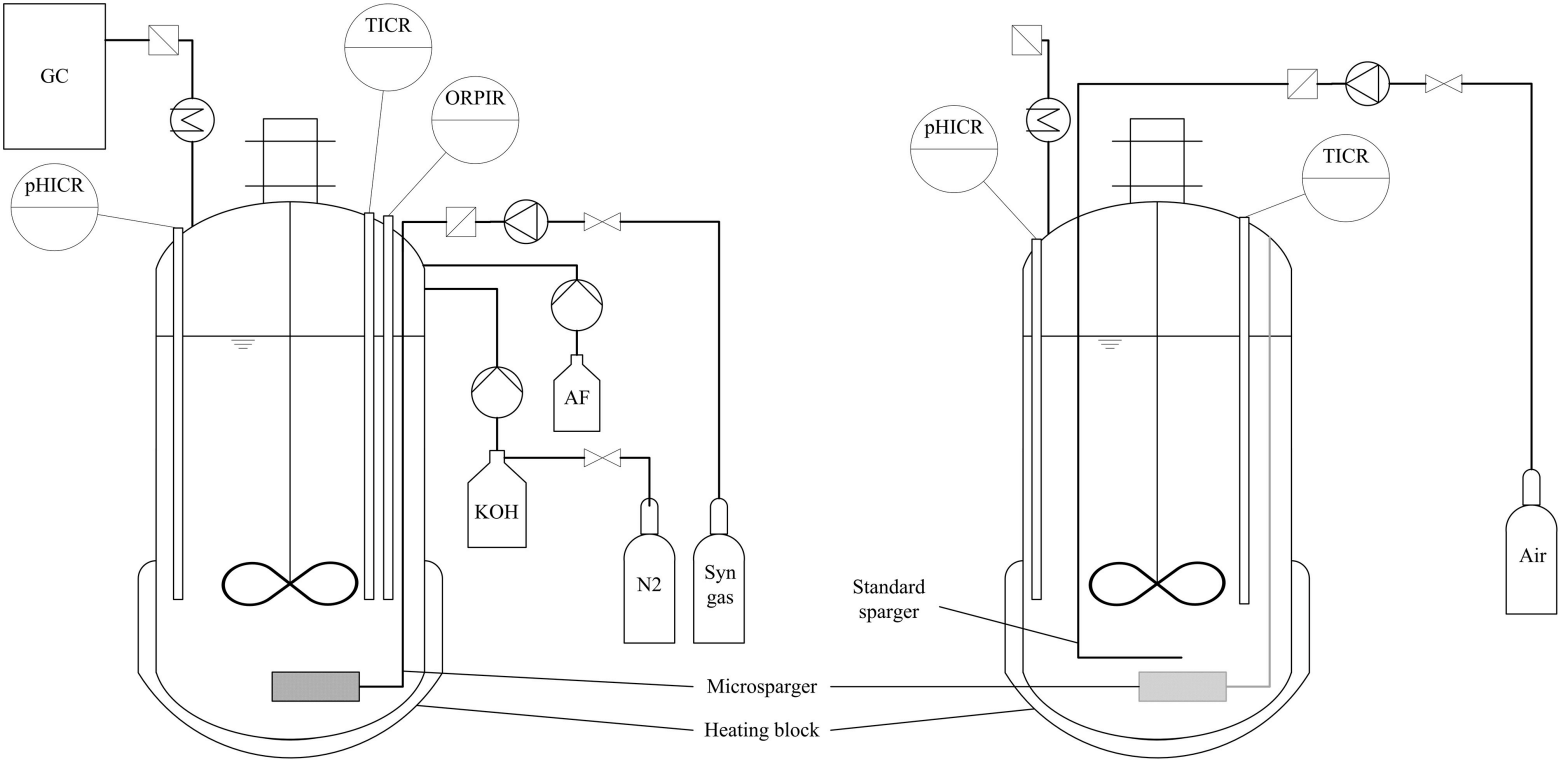
**Basic scheme of process setup for anaerobic syngas fermentation (left) and aerobic fungal fermentation (right)**. pHICR, pH indicate, control and record; TICR, temperature indicate, control and record; ORPICR, ORP indicate, control and record; AF, anti-foam; GC, gas chromatograph. For aerobic fungal fermentation the microsparger had to be turned sideways to make room for the standard sparger. No pH adjustment was conducted during fungal fermentation.

Fermentations were carried out in Minifors bench-top stirred tank reactors from Infors-HT (Switzerland) with a total volume of 2.5 L and a liquid volume of 1.5 L leaving a 1 L headspace. Figure 1 shows a basic scheme of the process setup for both anaerobic syngas fermentation and aerobic fungal fermentation.

For anaerobic syngas fermentation with *C. ljungdahlii*, each bioreactor was equipped with a Pt-100 temperature probe (Infors-HT, Switzerland), pH-probe (Mettler-Toledo, USA.) and an EasyFerm Plus ORP-probe (Hamilton, Switzerland) for measurement and recording of temperature, pH and redox potential during fermentation. The pH was regulated to 5.9 by addition of 4 M KOH solution which was kept under a nitrogen atmosphere to ensure anaerobic conditions. Temperature of the broth was maintained at 37 °C using the heating block of the bioreactor housing. To prevent foaming, bioreactors were fitted with a foam probe (Infors-HT, Switzerland) using Contraspum A 4050 HAC (Zschimmer und Schwarz, Germany) as an anti-foaming agent. Each bioreactor was equipped with a microsparger for creation of microbubbles to enhance mass transfer between gaseous and aqueous phase (Bredwell and Worden, 1998). The gas flow rate of 20 mL/min into the bioreactors was controlled using red-y smart series mass-flow-controller (MFC) from Vögtlin Instruments (Switzerland). Composition of the syngas used in this work was 32.5 vol-% H_2_, 32.5 vol-% CO, 16 vol-% CO_2_, and 19 vol-% N_2_ (Air Liquide, France), mimicking a composition of purified syngas from entrained bed gasification of straw. The headspace of the bioreactor was at atmospheric pressure. Gas-liquid mixing was achieved at 800 rpm using a stirrer setup for vortex formation (Figure 1). No baffles were used. Medium for bioreactor cultivations was prepared under aerobic conditions with the same composition as the maintenance- and pre-culture medium except for fructose which was omitted and cysteine-HCl which was reduced to 0.53 g/L. After autoclaving at 121 °C for 20 min the redox potential of the medium was lowered to about −200 mV by sparging with syngas and addition of the above stated amount of cysteine-HCl. Pre-cultures of *C. ljungdahlii* for reactor experiments were grown for 48 h with fructose as carbon and energy source. Bioreactors were inoculated with 10 % of their final volume using sterile silicon tubing with cannulas at both ends. After 96 h of growth on syngas the broth was either harvested for preliminary tests with *A. oryzae* or switched to aerobic conditions for the main coupling experiment.

For *A. oryzae* fermentation, 100 mL pre-culture medium was filled in 500 mL baffled shake flasks and inoculated with 2 × 10^7^ conidia. The culture was incubated for ca. 24 h at 30 °C and 100 rpm in a rotary shaker. Fungal pellets were washed twice with distilled water to remove pre-culture medium components before inoculation of the main culture. For shake flask cultivation, 100 mL of main-culture was transferred to 500 mL baffled shake flasks and mixed with 9 g sterile CaCO_3_. The flasks were inoculated with 10 vol-% of washed pre-culture and incubated at 120 rpm and 32 °C for 7 days. Fermentation in bioreactor was done in small scale bioreactor Minifors (vessel volume 2.5 L; Infors, Switzerland). To generate the conditions for malic acid production, some modifications of the reactor were necessary. The antifoam probe was replaced by a standard sparger to avoid clogging of the microsparger by the fungus and the redox potential probe was removed to enable inoculation with fungal preculture (fungal biomass of two preculture flasks) and CaCO_3_ was added (90 g per bioreactor). The microsparger was twisted sideways. Before inoculation, aeration was changed from syngas to air for ~30 min to remove all CO in solution and 200 μL of antifoam reagent (Contraspum A 4050 HAC, Zschimmer und Schwarz) was added. After inoculation, the fermentation took place at 35 °C with an aeration rate of 0.6 L/min and a stirrer speed of 300 rpm. Approximately every 24 h 5 mL samples were taken.

### Analytical methods

Measurement of the off-gas composition of the syngas fermentation was conducted via a GC-2010 Plus AT gas chromatograph (GC) system (Shimadzu, Japan) equipped with a customized column setup using a ShinCarbon ST 80/100 Column (2 m × 0.53 mm ID, Restek, Germany) and a Rtx-1 capillary column (1 μm, 30 m × 0.25 mm ID, Restek, Germany). The installed detector was a thermal conductivity detector with helium as the carrier gas. Column flow rate was 3 mL/min and oven temperature was kept at 40 °C for 3 min followed by a ramp of 35 °C/min. Total analysis time was 7.5 min. The off-gas line of every bioreactor was connected to the GC using a stream selector which, after each measurement, automatically selected the next bioreactor and thus enabled for automated off-gas analysis. Since the syngas in this work contained nitrogen and *C. ljungdahlii* is not able to use N_2_ as a nitrogen source we calculated the flow rate in the off-gas line (${\overset{\cdot }{V}}_{\text{off}\left(t\right)}$) according to

(1) $${\dot{V}}_{\text{off}(t)}\;=\frac{\varphi_{{\text{N}}_{2},\text{in}}}{\varphi_{{\text{N}}_{2},\text{off}}}{\dot{V}}_{\text{in}\left(t\right)}.$$

$$\begin{array}{l} {\dot{V}}_{\text{off}\left(t\right)}=\text{flowrate in off-gas line, mL/min}\\ \varphi_{{\text{N}}_{2},\text{in}}=\text{volume ratio of nitrogen in ingoing gas stream,-}\\ \varphi_{{\text{N}}_{2},\text{ off}}=\text{volume ratio of nitrogen in off-gas,-}\\ {\dot{V}}_{\text{in}\left(t\right)}=\text{flowrate of ingoing gas},\text{ mL/min} \end{array}$$

Using Equation (1), the ideal gas law and conditions in the lab (*T* = 298.15 K; *p* = 1.013 bar), it was possible to calculate the amount of substance flow rate ($\dot{n}$_i_) in mmol/min for each component i in the off-gas to

(2) $${\dot{n}}_{\text{i}}(t)\;=0.0409\;\frac{\text{mmol}}{\text{mL}}\;\varphi_{\text{i},\text{ off}}\;{\dot{V}}_{\text{off}(t)}.$$

$$\begin{array}{l} {\dot{n}}_{\text{i}(t)}=\text{amount of substance flow rate of substance i},\text{ mmol/min}\\ \varphi_{\text{i},\text{ off}}=\text{volume ratio of substance i in off-gas},\text{ –} \end{array}$$

Equation (3) calculates the amount of substance balance (${\Delta \overset{\cdot }{n}}_{\text{i}}$) between off-gas and gas inlet.

(3) $$\Delta {\dot{n}}_{\text{i}(t)}=\;{\dot{n}}_{\text{i},\text{in}(t)}\;-\;{\dot{n}}_{\text{i},\text{off}(t)}$$

$$\begin{array}{l} \Delta {\dot{n}}_{\text{i}(t)}=\text{amount of substance balance for substance i},\\ \text{ mmol/min}\\ {\dot{n}}_{\text{i},\text{in}(t)}\;=\text{amount of substance flow rate of i in ingoing gas}\\ \text{ stream},\text{ mmol/min}\\ {\dot{n}}_{\text{i},\text{off}(t)}=\text{amount of substance flow rate of i in off-gas},\\ \text{ mmol/min} \end{array}$$

Since there is no other sink or source for H_2_, CO, and CO_2_ other than the metabolism of *C. ljungdahlii* ${\Delta \overset{\cdot }{n}}_{\text{i}}$ equates to the uptake- or release rate of those molecules by the organisms.

To get the actual amount of CO that has gone into products and biomass it was necessary to account for CO that has been converted to CO_2_ by *C. ljungdahlii* and left the bioreactor. Therefore, the absolute value of Δ$\dot{n}$__CO__2__ was subtracted from Δ$\dot{n}$_CO_ if Δ$\dot{n}$__CO__2__ < 0 was true. Since $\dot{n}$_i, off_ was only determined in set intervals, linear interpolation of Δ$\dot{n}$_i_ between two points of measurement gave better approximation of the developing of Δ$\dot{n}$_i_. The total consumed amount of substance in the bioreactor at each time (*n*_i, R(*t*)_), that has gone into products and biomass, was then calculated by integration of the linear interpolation. This resulted in Equation (4).

(4) $$\begin{array}{l} n_{\text{i},\text{R}(t_{\text{j}})}=n_{\text{i},\text{R}(t_{\text{j}–1})}+\frac{\Delta {\dot{n}}_{\text{i}(t_{\text{j}})}-\Delta {\dot{n}}_{\text{i}(t_{\text{j–1}})}}{2}\left(t_{\text{j}}+t_{\text{j–1}}\right)+\\ \;\left(\Delta {\dot{n}}_{\text{i}(t_{\text{j–1}})}-\frac{\Delta {\dot{n}}_{\text{i}(t_{\text{j}})}-\Delta {\dot{n}}_{\text{i}(t_{\text{j–1}})}}{\left(t_{\text{j}}-t_{\text{j–1}}\right)}t_{\text{j–1}}\right)\left(t_{\text{j}}-t_{\text{j–1}}\right) \end{array}$$

$$\begin{array}{l} n_{\text{i},\text{R}(t)}=\text{consumed amount of substance in the bioreactor},\text{ mmol}\\ \;t\;=\text{process time},\text{ min} \end{array}$$

The complete derivation of equation (4) can be found in data sheet 1 in the supplemental data section. Dividing *n*_i, R(*t*)_ by the total amount of substance i that has gone into the bioreactor gave the ratio of fixation for each substance in percent as shown by Equation (5).

(5) $$E_{\text{i}(t)}=100\frac{n_{\text{i},\text{R}(t)}}{t\;{\dot{n}}_{\text{i},\text{in}(t)}}$$

$$E_{\text{i}(t)}\;=\text{ratio of fixation for substance i}$$

Liquid samples of 2 mL were collected every 2 h during the day. No samples were taken at night. Before the collection of a single liquid sample 3 mL of reactor broth were taken and discarded to account for the dead volume of the sampling line. Cell concentrations were determined using an Ultrospec1100pro spectrophotometer (Amersham Bioscience, USA) at a wavelength of 600 nm. Therefore, the optical density (OD) of 1 mL of a liquid sample was measured, then cells were removed via centrifugation at 16100 × g for 10 min and OD of the supernatant was measured. The difference of both values gave the OD of the sample. This procedure was necessary because OD values of the supernatant changed during fermentation. At measured OD > 0.45 the OD exceeded the linear range of the OD/cell mass relation and samples had to be diluted using 9 g/L NaCl solution. For correlation between OD and bio dry mass (BDM) the BDM at the end of the syngas fermentation was determined in duplicates. Therefore, 60 mL of fermentation broth were collected and transferred into pre-weight, dry screwing cap reaction tubes (30 mL each). The tubes were centrifuged at 4816 × g and 4 °C for 15 min. The supernatant was discarded and pellets were washed two times with a 9 g/L NaCl solution. The tubes with the washed pellets were dried at 60 °C for 48 h before they were weighted again and the BDM was calculated. The quotient of BDM and OD at the end of the syngas fermentation gave the BDM/OD correlation coefficient of 0.139 ± 0.041 g/L. Liquid samples were centrifuged to remove cells and the supernatant was stored frozen at −20 °C and used for further off-line analysis.

Measurement of fructose was done using an enzymatic D-fructose/D-glucose assay of Roche Yellow line (Hoffmann-La Roche, Switzerland) following the instructions delivered with the assay. Concentrations of ethanol and acetic acid of samples containing fructose were also measured with respective enzymatic assays from Roche Yellow line following their instructions. Samples which were collected after complete consumption of leftover fructose from the pre-cultures were analyzed for ethanol and acetic acid using a 6890N GC (Agilent, USA) equipped with auto-sampler, ROTICAP-FFAP capillary column (0.5 μm, 30 m × 0.32 mm ID, Carl-Roth, Germany) and flame ionization detector. Carrier gas was helium with a pressure of 1 bar and split ratio was 7.5:1. Analytical standard mixture consisted of 5 mM ethanol, 5 mM sodium acetate, and 9.09 mM isobutanol in 0.18 M HCl. Samples were prepared by acidifying 500 μL of sample with 50 μL internal standard solution consisting of 100 mM isobutanol in 2 M HCl. Analysis was conducted by injecting 1 μL of sample or standard. The temperature profile of the column oven started with initial 60 °C for 2 min followed by a temperature ramp of 10 °C/min up to an end temperature of 180 °C. Total analysis time was 20 min.

The concentrations of malic and acetic acid during cultivation with *A. oryzae* were quantified with HPLC. Fermentation broth samples were pretreated and analyzed as described in Ochsenreither et al. (2014). To resolve as calcium salt precipitated organic acids, 1 mL of well-mixed sample was mixed with 1 mL of 3 M H_2_SO_4_ and 3 mL of distilled water and incubated at 80 °C for 20 min. One milliliter of the mixture was transferred to a 1.5 mL Eppendorf tube and centrifuged in a bench top centrifuge for 5 min at 20,000 × g. The supernatant was used for HPLC analysis. The analysis was performed at 30 °C with a standard HPLC device (Agilent 1100 Series, Agilent, USA) prepared with a 15 cm reversed phase column (Synergi™4 μm Fusion-RP 80 Å, LC Column 150 × 4.6 mm, Phenomenex, Germany). Mobile phase solution A was 100 % methanol, and solution B was 20 mM KH_2_PO_4_, pH 2.5. The flow rate was 1 mL/min and a gradient was used for the separation of organic acids: 0–0.5 min 100 % eluent B, 0.5–10 min increase of eluent A from 0 to 10 %,10–12 min a decrease of eluent A from 10 back to 0 %, and 12–14 min again 100 % eluent B. The injection volume was 10 μL and the detection was performed by a UV detector at 220 nm. Malic acid standard was purchased from Sigma-Aldrich (Germany), acetic acid standard from Carl-Roth (Germany). Both were used for peak identification and calibration. The linear detection range went from 0.1 g/L to 5 g/L malic acid and acetic acid.

## Results

### Preliminary experiments

#### Optimization of bioreactor medium for sequential production of malic acid from acetic acid

Since malic acid is produced by *A. oryzae* only under nitrogen limited conditions (Knuf et al., 2013) it was necessary to reduce the ammonia content of the medium to ensure nitrogen limitation after syngas fermentation. Initial cultivations, using the above mentioned medium for syngas fermentation, were conducted to determine the amount of ammonia consumed by *C. ljungdahlii* during 96 h of growth on synthesis gas (see Table 1). Based on the consumed amount of ammonia, the NH_4_Cl content of the medium for all following pre-cultures and bioreactor experiments was reduced to 0.33 g/L. Ion chromatography for ${\text{NH}}_{4}^{+}$-detection was kindly conducted by the section for Bioprocess Engineering of the Institute of Process Engineering in Life Sciences, KIT. Results of medium optimization and preliminary cultivations with NH_4_-reduced medium are shown in Table 1. Measurement of ammonia concentration after 96 h of syngas fermentation using NH_4_-reduced medium was not possible due to high amounts of potassium ions in the broth.

**Table 1:** **Results of preliminary experiments with ***C. ljungdahlii*** growing with syngas as sole carbon source**.

**Medium**	**^*c*^NH_4_ Cl g/L**	**^*c*^BDM g/L**	**^*c*^acetic acid g/L**	**^*c*^EtOH g/L**	**^Δ*c*^NH_4_Cl g/L**	**^*c*^H_2_, R mol/L**	**^*c*^CO, R mol/L**	**^*c*^CO_2_, R mol/L**	**^*Y*^P∕S g/g**
Syngas fermentation^a^	2.5	0.76 ± 0.29	15.27 ± 1.68	0.57 ± 0.58	0.39 ± 0.06	0.75 ± 0.04	0.77 ± 0.05	0.01 ± 0.04	0.68 ± 0.05
NH_4_-reduced^b^	0.33	0.45 ± 0.14	17.08 ± 2.28	1.14 ± 0.77	N/A	0.64 ± 0.05	0.74 ± 0.04	−0.09±0.13	0.67 ± 0.04

a three replicas,

b *six replicas, c__H__2_, R_, consumed amount of hydrogen per liter reactor volume; c_CO, R_, consumed amount of carbon monoxide per liter reactor volume; c_C_O__2_, R_, consumed amount of carbon dioxide per liter reactor volume, N/A: not available*.

After 96 h of fermentation on syngas using syngas fermentation medium (see above) with 2.5 g/L ammonia chloride, *C. ljungdahlii* consumed 386.7 mg/L ammonia. Nevertheless, fermentation under nitrogen reduced conditions yielded an average of 17.08 g/L acetic acid and 1.14 g/L ethanol compared to 15.27 g/L acetic acid and 0.57 g/L ethanol when *C. ljungdahlii* was cultivated with an excess of ammonia. In ammonia rich medium *C. ljungdahlii* consumed 0.75 mol/L of hydrogen and 0.77 mol/L of carbon monoxide and in NH_4_-reduced medium they consumed 0.64 mol/L of hydrogen and 0.74 mol/L of carbon monoxide. The values for consumed carbon dioxide were around zero for all fermentations in syngas fermentation medium with an average of 0.01 mol/L whereas in NH_4_-reduced medium in some cultivations the bacteria released CO_2_ to the off-gas, thus the negative mean value of −0.09 mol/L. For both medium types the ratio of products (acetic acid and ethanol) to consumed substrates (*Y*_P∕S_) is roughly the same, with averages of 0.68 g/g for syngas medium and 0.67 g/g for NH_4_-reduced medium.

#### Preliminary experiment for fungal fermentation

Large quantities of organic acids are produced by certain fungi generally under nitrogen limiting conditions and a simultaneous excess of carbon source. For the production of malic acid with *A. oryzae* these requirements are met in a special production medium as published by Battat et al. (1991). However, this production medium is considerably different from the syngas fermentation medium, a general microbial cultivation medium. Therefore, preliminary experiments were conducted to determine the suitability of acetic acid and ethanol as carbon source, which was never shown before, and the influence of other medium ingredients on malic acid production. These experiments are summarized in Table 2.

**Table 2:** **Results of preliminary experiments with ***A. oryzae*** regarding the influence of different medium components and carbon sources on malic acid production**.

**Medium**	**^*c*^Carbon source g/L**	**Modification/Pretreatment**	**^*c*^Malic acid g/L**	**Y^b^_P/S_ g/g**
Organic acid production medium^a^	Glucose 109	–	47.84 ± 3.49	0.8
Organic acid production medium	Glucose 109	0.533 g/L Cysteine	44.2 ± 5.85	0.64
Organic acid production medium	Glucose 109	0.533 g/L Sodium sulfide	54.04 ± 14.16	0.65
Organic acid production medium	Acetic acid 50	Exchange of carbon source	8.62 ± 1.15	0.28
Organic acid production medium	Acetic acid, ethanol 33.33, 16.66	Exchange of carbon source	11.68 ± 1.27	0.55
Organic acid production medium	Ethanol 50	Exchange of carbon source	0	0
Syngas fermentation medium^a^	Acetic acid 50	Exchange of carbon source	2.69 ± 0.81	0.09
Syngas fermentation medium	Acetic acid 50	Exchange of carbon source, without ammonium	4.11 ± 0.50	0.37
Syngas fermentation medium: fermented	Acetic acid 9.80 ± 0.21	Removal of *C. ljungdahlii* biomass	0	0
Syngas fermentation medium: fermented	Acetic acid 15.84 ± 1.55	Reduced ammonium	4.34 ± 0.10	0.27
Syngas fermentation medium: fermented	Acetic acid 8.88 ± 3.42	Reduced ammonium Removal of *C. ljungdahlii* biomass	0	0

a control approach,

b yield is given after 168 h cultivation (Y_P/S_).

*All concentrations are given as average of three independent experiments ± standard deviation*.

#### Evaluation of carbon source and medium components

**Figure 2:**
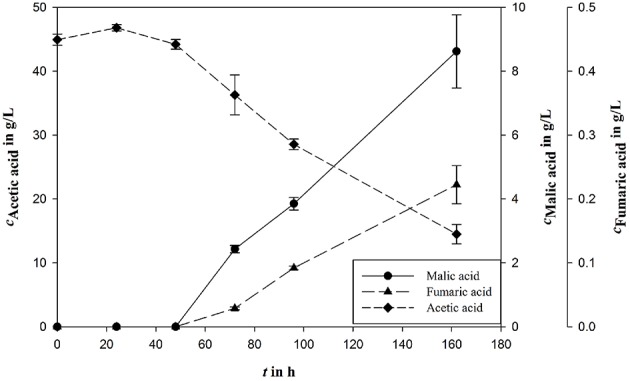
**Malic acid production (***c***_***Malic acid***_) using acetic acid (***c***_***Acetic acid***_) as carbon source in fungal organic acid production medium**. Fumaric acid (*c*_*Fumaric acid*_) is the most important side product during *A. oryzae* fermentation. All concentrations are given as average of three independent experiments ± standard deviation.

Influence of major medium components on malic acid production was evaluated in shake flask experiments and fermentation experiments in bioreactors as indicated. The biggest differences of the established process were the carbon source and the nitrogen concentration. It could be shown that acetic acid is an appropriate carbon source for malic acid production in established malic acid production medium with an *Y*_P∕S_ of 0.28 g/g and a final product concentration of 8.62 ± 1.15 g/L (Figure 2).

The most important byproduct of syngas fermentation is ethanol. Various concentrations of both, acetate and ethanol, could be achieved in different fermentations. To analyze the influence on or the suitability of these molecules as substrates for malic acid production, ethanol was added to production medium. With a yield of 0.55 g/g the acetic acid/ethanol-approach reached a final product concentration of 11.68 ± 1.27 g/L (Table 2). When ethanol was used as sole carbon source, acid production was not detected. In the second round of experiments the reduction agents cysteine and sodium sulfide were tested. These agents are used during syngas fermentation to reduce contaminating oxygen. Because cysteine is also a potential nitrogen source for *A. orzyae*, tests of the effects on malic acid production are necessary. The results showed a minor influence of the preferred reduction agent cysteine with a final malic acid concentration of 44.2 ± 5.85 g/L (*Y*_P∕S_ of 0.64 g/g), as well as the alternative reduction agent sodium sulfide with a *Y*_P∕S_ of 0.65 g/g (54.04 ± 14.16 g/L) compared to the control approach, where no modifications were done, with 47.84 ± 3.49 g/L where a higher *Y*_P∕S_ of 0.8 g/g could be achieved (Table 2).

It could be shown that the reduction agents are not problematic and acetic acid is an appropriate carbon source for malic acid production. The syngas fermentation medium, which has a significant different composition compared to the malic acid production medium, was a further challenge. Especially the initial ammonium concentration proved to be problematic as ammonium was not completely consumed during syngas fermentation, so that considerable amounts of ammonium remained for the subsequent fungal fermentation medium. Therefore, to prove if syngas fermentation medium is in general suitable for malic acid production, fungal cultivations in shake flasks with normal concentration and without nitrogen were conducted. Syngas fermentation medium was mixed, autoclaved and enriched with acetic acid as carbon source. Normal ammonium concentration leads to a final malic acid concentration of 2.69 ± 0.81 g/L with a yield of 0.09 g/g. If no nitrogen source was added 4.11 ± 0.50 g/L and a yield of 0.37 g/g could be achieved (Table 2). Therefore, the syngas fermentation medium is a suitable medium for malic acid production, but nitrogen concentration must be limited to a minimum level.

**Figure 3:**
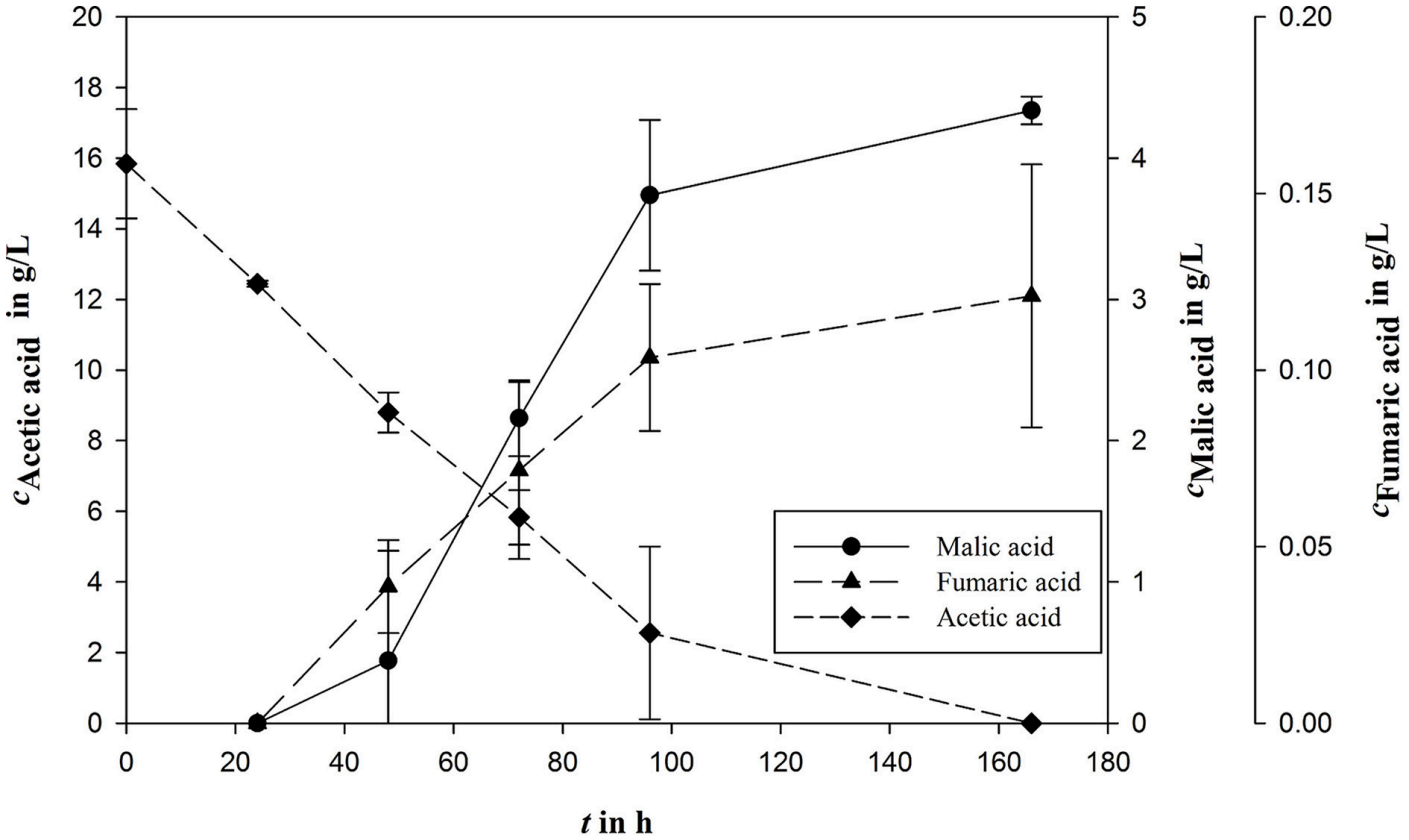
**Malic acid production (***c***_***Malic acid***_) using acetic acid (***c***_***Acetic acid***_) as carbon source in fermented syngas fermentation medium**. Fumaric acid (*c*_*Fumaric acid*_) is the most important side product during *A. oryzae* fermentation. All concentrations are given as average of three independent experiments ± standard deviation.

However, in the sequential mixed culture fermentation, the syngas fermentation medium might possibly undergo unknown modifications as a result of *Clostridia* cultivation. To analyze the effects to the *A. oryzae* fermentation, an authentic already fermented medium was used including *Clostridia*-produced acetic acid. On the one hand, the medium containing biomass is a possible nitrogen source; on the other hand biomass could contain some important medium ingredients. To test the influence of biomass on malic acid production, bacterial biomass was either removed by centrifugation or left inside in nitrogen rich or reduced medium. For the first experiments in the bioreactor, a nitrogen rich medium was used. To reduce the nitrogen concentration, biomass was removed by centrifugation. In this approach, no malic acid production could be measured. Further tests in shake flasks were done with ammonium reduced medium, with either removed or not removed biomass. With microbial biomass a malic acid concentration of 4.34 ± 0.10 g/L was produced from 15.84 ± 1.55 g/L acetic acid corresponding to a yield of 0.27 g/g (Figure 3). The removal of microbial biomass prevented product formation.

Because initial shake flask experiments were promising, the sequential mixed culture approach was tested under realistic conditions, i.e., syngas fermentation followed by fungal fermentation without medium removal and/or delay in between.

### Coupling experiment for sequential production of malic acid from acetic acid

#### Syngas fermentation

**Figure 4:**
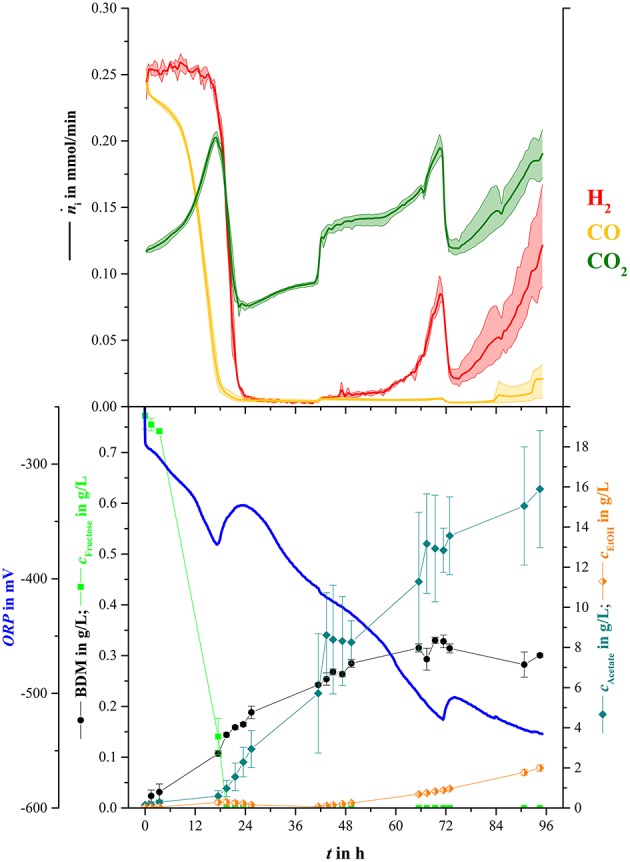
**Mean online and offline values for syngas fermentation part of sequential mixed culture (three replicas)**. Upper part: Average amount of substance flow rates for hydrogen (red), carbon monoxide (yellow), and carbon dioxide (green) in the off-gas. The lightly colored areas around the average lines show minimum and maximum variance between bioreactors. Bottom part: Average values for *ORP* (blue line), BDM (black dots), fructose concentration (light green squares), acetate concentration (blue-green diamonds), and ethanol concentration (orange half-filled diamonds).

For the main experiment, NH_4_-reduced medium was used for syngas fermentation to ensure nitrogen limited conditions after 96 h. Syngas was delivered into the broth with a starting rate of 20 mL/min and was increased after 42 h to 25 mL/min. Figure 4 shows mean values for offline measured concentrations of biomass, fructose, acetate, and ethanol as well as for online measured values for amount of substance flow rates of hydrogen, carbon monoxide, and carbon dioxide in the off-gas.

In the beginning of the experiment, fructose concentration and amount of carbon monoxide in the off-gas decreased constantly until fructose was not detectable anymore at 19.5 h. Biomass concentration continuously increased until 49 h and stayed at 0.3 g/L for the rest of the fermentation. Acetate and Ethanol concentrations increased to maximum mean values at the end of the syngas fermentation of 15.9 g/L and 2.0 g/L, respectively. Similar to the decrease of carbon monoxide in the off-gas, carbon dioxide increased up to a local maximum of 0.2 mmol/min after 17.3 h of cultivation, then dropped to an average of 0.07 mmol/min when hydrogen consumption started. Hydrogen off-gas values stayed as low as 0.005 mmol/min and slightly increased when the rate of ingoing syngas was increased. After about 47 h the hydrogen content in the off-gas started to increase. In contrast to hydrogen and carbon dioxide, carbon monoxide values in the off-gas stayed low until 83.0 h when they started to increase until the end of the fermentation. The decreases of gas flow rates at 71.0 h were due to reduction of the ingoing gas stream to 20 mL/min.

**Figure 5:**
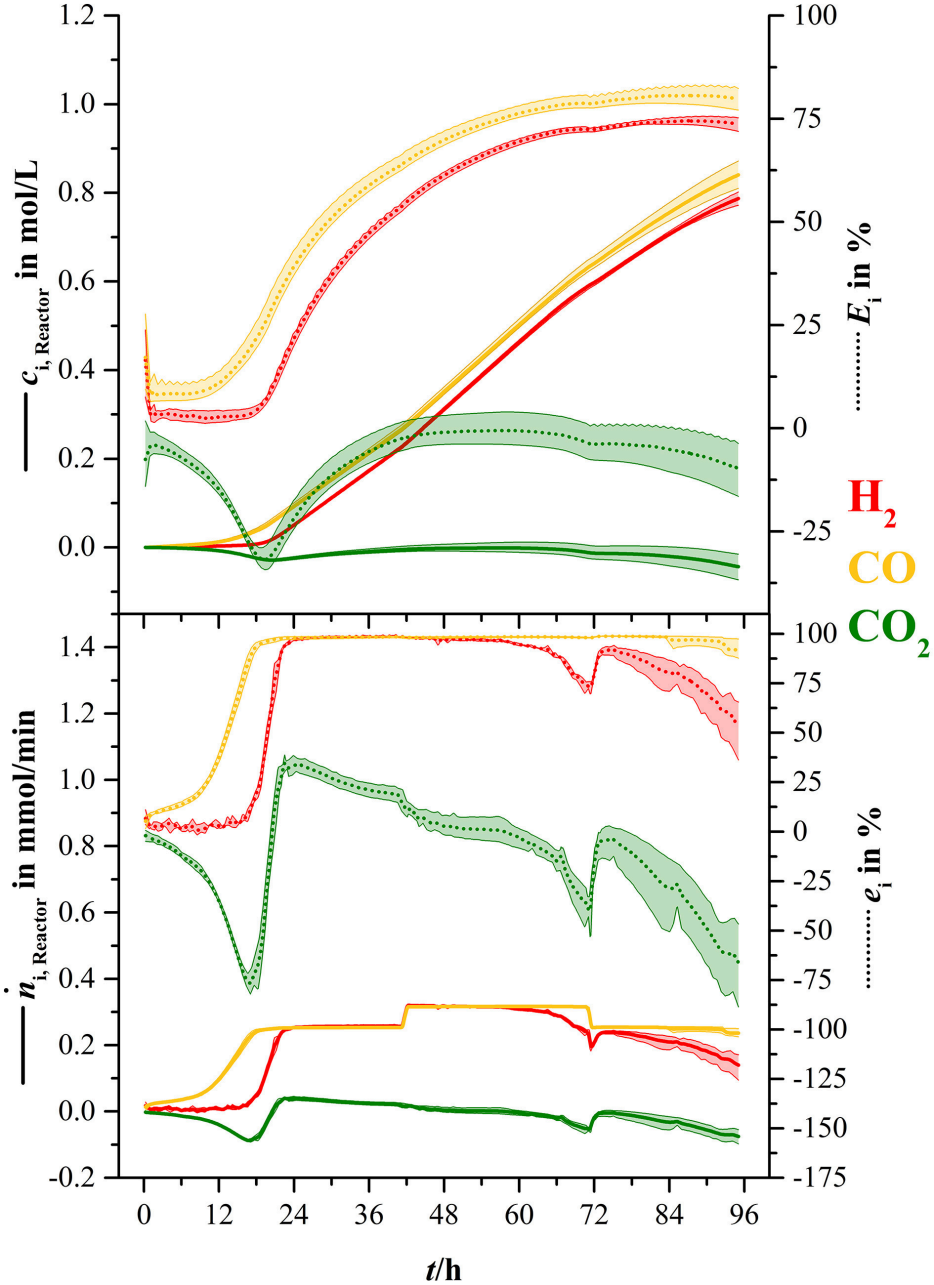
**Overall concentration of consumed substrates and consumption rate for hydrogen (H_**2**_, red), carbon monoxide (CO, yellow), and carbon dioxide (CO_**2**_, green)**. Values are shown in absolute values *c*_i, R(*t*)_ and ${\Delta \overset{\cdot }{n}}_{\text{i}}$ (solid lines) and in percent of the amount gone into the bioreactor at each specific time (*E*_i_, dotted lines) and in percent of the rate of the ingoing amount of substance (*e*_i_, dotted lines). The lightly colored areas around the average lines show minimum and maximum variance between three bioreactors.

For better illustration, the consumed amount of substance (*n*_i, R_) of each syngas component (except nitrogen) and the rates ($\Delta {\overset{\cdot }{n}}_{\text{i}}$) of consumption were calculated over the course of this fermentation. Figure 5 shows the results of those calculations.

The amount of consumed carbon monoxide increased continuously from inoculation and reached an average maximum of 0.84 mol/L. This equals to 79.6 % of the total CO that went into the bioreactor (dotted yellow line). The amount of consumed hydrogen started to increase considerably after 18.5 h and went up to 0.77 mol or 73.6 % of total hydrogen (dotted red line). Similar to the increase of carbon dioxide in the off-gas in Figure 4, the amount of consumed carbon dioxide decreased down to −0.03 mol/L or −31.8 % of the amount of carbon dioxide at 19.4 h. From that point, the amount of consumed carbon dioxide increased to 0 mol/L and started to decrease down to −0.05 mol/L (−9.8 %) when hydrogen consumption faded. Uptake rate of carbon monoxide increased continuously during the first 20.2 h, where it reached its maximum average of 0.25 mmol/min equaling 98 % of the ingoing carbon monoxide stream at that time. Hydrogen uptake rate started to increase at 15.5 h and reached a maximum average of 0.25 mmol/min or 98 %. At 41.5 h the uptake rate of carbon monoxide and hydrogen increased to 0.3 mmol/min due to the elevated gas sparging rate. After 48 h the hydrogen uptake rate started to decrease and went down to 0.14 mmol/min at the end of the fermentation.

Directly following the syngas fermentation the reactor was changed to fungal fermentation as stated above. Microbial biomass was not removed.

#### Sequential mixed culture

**Figure 6:**
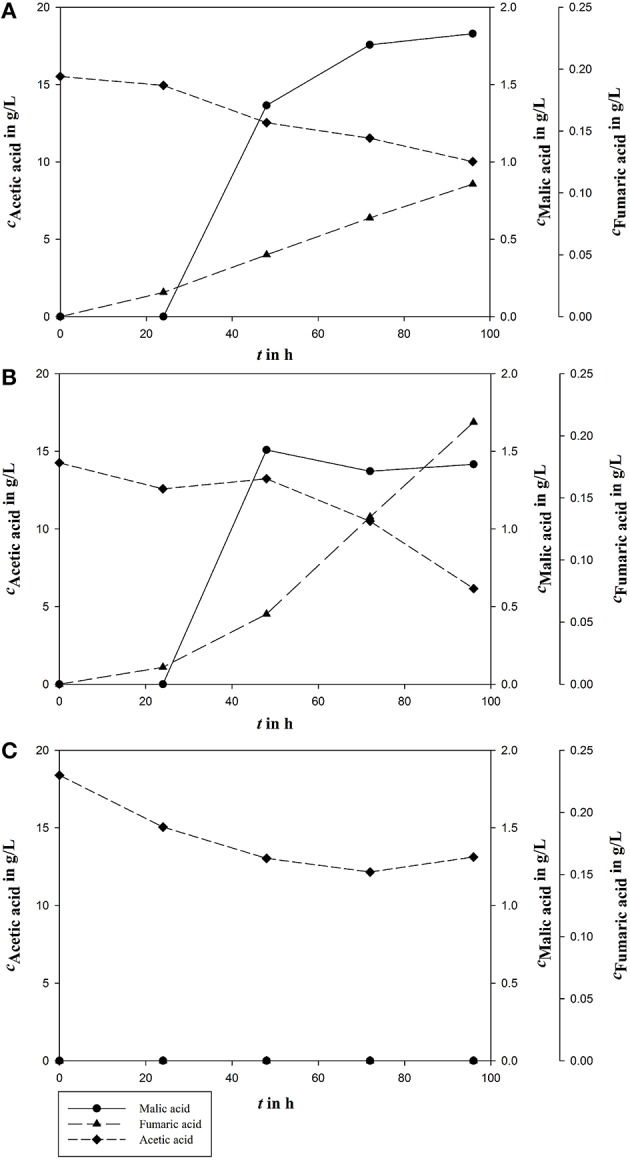
**Malic acid production (***c***_***Malic acid***_), and acetic acid (***c***_***Acetic acid***_) consumption in the three bioreactors A, B, and C from triple approach for syngas fermentation after 96 h of fermentation**. Fumaric acid (*c*_*Fumaric acid*_) is the main side product during *A. oryzae* fermentation.

The sequential mixed culture was accomplished in three replicates in the described fermentation setup in a bioreactor. Results for malic acid fermentation are shown in Figure 6.

In two of the three bioreactor runs, malic acid production was detected. In one bioreactor, acetic acid was partly metabolized, but no product was formed. In reactor **A** acetic acid decreased from 15.53 g/L to 10.02 g/L during malic acid production of 1.83 g/L corresponding to a yield of 0.33 g/g. In reactor **B**, the initial acetic acid concentration of 14.26 g/L was reduced during fermentation process to 6.15 g/L by simultaneous production of 1.42 g/L malic acid resulting in a yield of 0.18 g/g. During cultivation in reactor **C**, 5.39 g/L acetic acid was metabolized in total, from initially 18.39 g/L to 13.12 g/L, but without product formation. After 96 h of fermentation, the three reactors differed greatly. In reactor **A** the malic acid concentration decreased to 0. In reactor **B** malic acid concentration increased to 2.02 g/L and in reactor **C** further no malic acid production could be observed (data not shown).

## Discussion

For the sequential mixed culture fermentation from syngas to malic acid, the main challenges were the requirements of the involved microorganisms in terms of reactor set-up, medium composition and product synthesis. Optimizing product yield and productivity for a certain process usually addresses the needs of the organism involved. Since sequential mixed culture fermentation uses at least two different organisms the key aspect for sequential mixed culture fermentation is either a medium compromise for both organisms or the compatibility of the first (optimized) medium for the second organism in terms of product synthesis. Furthermore, the second organism has to be able to use the product of the first process as a carbon source. The combination of both aspects must be fulfilled to achieve an optimal value added chain from syngas to malic acid.

For the first time, this study shows that *A. oryzae* is able to use acetic acid, the main product of syngas fermentation, as a proper carbon source for malic acid production. The metabolic pathways and mechanisms are largely understood, when using carbohydrates as carbon source. A partly reductive TCA cycle following on glycolysis and malic acid is synthesized from pyruvic acid and oxaloacetic acid to malic acid (Osmani and Scrutton, 1983; Peleg et al., 1988, 1989; Bercovitz et al., 1990). Fermentative mechanisms for malic acid production from other carbon sources are still not fully known and therefore speculative. For growing on acetic acid, acetyl-CoA synthetase (ACS) was described as a key enzyme for the metabolism of ethanol and acetic acid, which converts acetic acid to acetyl-CoA in *C. albicans* (Carman et al., 2008). Acetyl-CoA may then enter the glyoxylate cycle which is partly located in the peroxisome and is done for gluconeogenesis. Malic acid occurs in this pathway as an intermediate. This process may be the pathway for malic acid production from acetic acid. This metabolic flux is summarized by Strijbis and Distel (2010).

### Preliminary experiments with *C. ljungdahlii*

Cultivation of *C. ljungdahlii* in medium containing 2.5 g/L ammonia chloride for 96 h resulted in consumption of 0.131 g/L ammonia. This equals to a concentration of 0.39 g/L ammonia chloride. To ensure that after 96 h of syngas fermentation no ammonia is left, a total of 0.5 g ammonia chloride was used for 1.5 L of medium. The results of preliminary experiments shown in Table 1 indicate that reducing the ammonia concentration in the medium does not negatively affect product formation, substrate consumption and overall product yield, although biomass concentration is slightly lower in NH_4_-reduced medium. This is consistent with experimental data from Xu et al. (2011) reporting slight differences in biomass concentration in this range of ammonia concentrations.

### Preliminary experiments with *A. oryzae*

Comparing the fungal fermentation on acetic acid in the two different media, it could be seen that a reduction of the nitrogen concentration is mandatory. If ammonium is omitted in syngas fermentation medium a similar yield was achieved for malic acid production medium (*Y*_P∕S_ of 0.28 g/g) and syngas fermentation medium (*Y*_P∕S_ of 0.37 g/g) using acetic acid despite the nitrogen present in malic acid production medium. Although observed yields were similar, malic acid concentration in syngas fermentation medium is approximately half as high as in optimized organic acid production medium (8.62 ± 1.15 g/L against 4.11 ± 0.50 g/L) after 168 h. There is also a lack in malic acid production depending on presence of microbial biomass. In medium, containing microbial biomass and with reduced ammonium, malic acid production was firstly detected after 72 h of cultivation. If microbial biomass was not removed malic acid concentration reached the detection limit already after 48 h. Because biomass itself could be used as source of minerals, nutrients, and vitamins, it might be helpful for the adaption of the fungus to acetic acid as carbon source. Nevertheless, for this sequential mixed culture approach it is a good result that biomass has a positive effect on malic acid production and does not need to be removed. A further challenge was the side product of syngas fermentation, ethanol. As reported in several studies, various concentrations of this alcohol could be produced during cultivation of acetogenic bacteria. With *C. ljungdahlii* ethanol concentrations of 48 g/L could be achieved using syngas from coal as energy and carbon source (Klasson et al., 1993). Our experiments with ethanol as sole carbon source for fungal fermentation showed no malic acid production, so that it can be assumed that ethanol alone in those high concentrations is not a suitable carbon source for *A. oryzae*. However, the acetic acid/ethanol mixed approach showed the highest yield for malic acid compared to the other acetic acid fermentations (*Y*_P∕S_ of 0.55 g/g). It was shown that stress conditions for the fungus are beneficial for malic acid production, due to an up-regulation and overexpression of the genes involved in the malic acid production pathway (Knuf et al., 2013). Ethanol as a solvent may lead to stress for *A. oryzae* during cultivation which enhances the product synthesis. It is also possible that ethanol in low concentrations could be metabolized and serves as possible carbon source for malic acid production on the described pathway. In this case the ethanol amounts, produced in this process (0.75–1.14 g/L) would not be problematic. All in all the results of preliminary experiment led to the assumption that a sequential mixed culture from syngas to malic acid is a promising approach.

### Sequential mixed culture from syngas to malic acid

During the first 18 h of syngas fermentation *C. ljungdahlii* was co-consuming fructose and carbon monoxide but did not consume hydrogen. The reason for this could be that CO is a known inhibitor of hydrogenase activity in Clostridia (Gray and Gest, 1965; Chen and Blanchard, 1978; Kim et al., 1984; Devarapalli et al., 2016). Thus, hydrogen consumption can only start once the carbon monoxide partial pressure in the broth is low enough. Considerable acetate formation was detectable after consumption of hydrogen started. When the fructose in the medium is depleted and hydrogen consumption starts, the organisms begin to significantly produce acetate. This point was indicated by a temporary increase of the redox potential in the broth.

Up to the time point when hydrogen consumption started to decrease, acetate was continuously produced. This point also marked the beginning of ethanol formation. In case the decrease of hydrogen consumption was due to the increase in the gas sparging rate and therefore the carbon monoxide supply, we reduced the flow rate of syngas to stabilize the uptake rates again. This brought a temporary improvement but hydrogen consumption rate decreased for the rest of the syngas fermentation. The observed occurrence of sudden decrease in hydrogen consumption is a known phenomenon when cultivating *C. ljungdahlii* on syngas as can be seen in Cotter et al. (2009) and Maddipati et al. (2011). However, there is nothing known about why hydrogen consumption decreases.

For the main experiment, average acetate concentration after 96 h was about 1 g per liter lower and standard deviation was 0.5 g per liter higher compared to preliminary bioreactor experiments. The differences in acetate concentration between the three bioreactors might be due to different rates of decreasing hydrogen consumption. In addition, off-gas composition of the bioreactors for H_2_, CO, and CO_2_ showed increasing deviations after the reduction of the ingoing gas stream.

The malic acid production in the three bioreactors with *A. oryzae* varied widely. Bioreactor **A** and **B** showed both malic acid production after 48 h as expected from shake flask experiments. The curve of organic acid production is similar, but reactor **B** had already reached a plateau after 48 h, whereas reactor **A** reached the plateau after 96 h. There is also a spread of 0.15 g/L of the yield between bioreactor **A** and **B**. The reason for the lack of malic acid production in reactor **C** and the different yields in both other reactors is very difficult to discuss due to the high complexity of the medium composition after syngas fermentation. It seems that small differences in syngas fermentation may have large effects on the following fungal fermentation. Because of that fact, this should not be seen as a triplicate but as three different batches from syngas fermentations. Despite, the fact that interpretation of the results is difficult and the failure in one bioreactor it was clearly shown that malic acid production from syngas by sequential mixed culture fermentation is possible. The overall conversion efficiency of syngas into acetate and ethanol for the syngas fermentation part can be expressed as *Y*_P∕S_ of 0.66 g acetic acid and ethanol per gram of consumed syngas. Combined with the *Y*_P∕S_ for the conversion of acetic acid into malic acid we achieved an overall *Y*_P∕S_ for the conversion of CO and H_2_ into malic acid of 0.22 g/g (3.5 g malic acid per mol syngas consumed) for reactor **A** and 0.12 g/g (1.9 g malic acid per mol syngas consumed) for reactor **B**. This was achieved with complete conversion of CO and H_2_ into products. Since there are no reported processes for production of malic acid from syngas we compare our yields with anaerobic production of other C_4_ molecules. Anaerobic processes for production of butanol from sugars described in literature gave *Y*_P∕S_ values between 0.1 g/g and 0.3 g/g when using *C. beijerinckii* or *C. acetobutylicum* and sugar from lignocellulosic substrates as a carbon source (Schiel-Bengelsdorf et al., 2013). Using syngas for production of butanol as did Lewis et al. (2007) yielded 0.08 g butanol per gram of consumed carbon monoxide. Other processes for production of C_4_-molecules using anaerobic organisms and syngas as a substrate described in literature do not state *Y*_P∕S_ values which prevents proper comparison.

Although sequential mixed cultures have been used for centuries in food industry, e.g., sake production, applications for production of value added chemicals is rare. It could be shown, that this kind of biotechnological process is suitable for the production of low price chemicals like single cell oils for biofuel production (Hu et al., 2016). There are also some approaches for interlinking cultivations, like co-cultivating a homoacetogen (e.g., *C. ljungdahlii*) and an anaerobic organism that is able to grow on syngas, ethanol or acetate and produces butyrate or butanol as described by Datta and Reeves (2014), a combination of algae and yeast fermentation (Dillschneider et al., 2014), dextran fermentation (Kim and Day, 1994) and biogas production, but sequential fermentations are rare.

## Conclusion

We could successfully show that production of high-value L-malate from syngas is possible. Further increase of yield is feasible as the process medium was neither optimized for acetic acid production nor for malic acid production and only wild type strains of *C. ljungdahlii* and *A. oryzae* were used. Both strains are available at the DSMZ. The advantage of this kind of biotechnological process is the extension of the product portfolio of anaerobic syngas fermentation. Because of the toxicity of oxygen to *Clostridia*, there is no further step necessary than changing the sparging from syngas to air, to prepare the medium for fungal fermentation. The work at hand demonstrates that *A. oryzae* is able to use acetic acid as a substrate for malic acid formation. Moreover it shows that it is possible to link anaerobic syngas fermentation and aerobic malic acid production using sequential mixed cultures of *C. ljungdahlii* and *A. oryzae*. In doing so we not only broadened the feedstock for malic acid production from glycerol and sugars to the whole feedstock of gasification processes but also reported the highest yields to date for the production of C_4_ components from syngas.

## Author contributions

FO: Substantial performance of the experiments with *C. ljundahlii* and the anaerobic part. Writing, as co-first author, the syngas part of the manuscript and parts of introduction and Discussion. SD: Substantial performance of the experiments with *A. oryzae* and the aerobic part. Writing, as co-first author, the fungal part of the manuscript and parts of introduction and Discussion. SD and FO are co-first authors and contributed equally to the paper. NV: Substantial performance of the experiments with *A. oryzae* and the aerobic part. MZ: Substantial performance of the experiments with *C. ljundahlii* and the anaerobic part. AN: Substantial contribution to the conception of the syngas fermentation and critically revising of the final version to be published. KO: Substantial contribution to the conception of the fungal fermentation and critically revising of the final version to be published. CS: Substantial contribution to the idea of the coupling fermentation and critical revision of the work for important intellectual content.

### Conflict of interest statement

The authors declare that the research was conducted in the absence of any commercial or financial relationships that could be construed as a potential conflict of interest.
