# Supplementary material for: Sequential Mixed Cultures: From Syngas to Malic Acid
Source: Front Microbiol. 2016 Jun 21;7:891. doi: 10.3389/fmicb.2016.00891 (PMC4914491; doi:10.3389/fmicb.2016.00891)
Supplement: Supplementary file 1 [file DataSheet1.PDF]

Derivation of equation 4

Amount of substance balance per minute between off-gas in gas inlet $[\Delta\dot{n}_i] = \frac{\text{mmol}}{\text{min}}$

$$\Delta\dot{n}_{\text{i}} = \dot{n}_{\text{i}} - \dot{n}_{\text{i,in}}$$

Linear interpolation of $\Delta\dot{n}_{\text{i}}$ between two points of measurement for better approximation using point-slope-form:

$$\Delta\dot{n}_{\text{i},(t)} = \frac{\Delta\dot{n}_{\text{i},(t_{\text{j}})} - \Delta\dot{n}_{\text{i},(t_{\text{j}-1})}}{t_{\text{j}} - t_{\text{j}-1}} t + \left(\Delta\dot{n}_{\text{i},(t_{\text{j}-1})} - \frac{\Delta\dot{n}_{\text{i},(t_{\text{j}})} - \Delta\dot{n}_{\text{i},(t_{\text{j}-1})}}{t_{\text{j}} - t_{\text{j}-1}} t_{\text{j}-1}\right) \text{ for } t_{\text{j}-1} \leq t \leq t_{\text{j}}$$

Integration of $\Delta\dot{n}_{\text{i},(t)}$ from $t_{\text{j-1}}$ to t_{j} gives the total consumption of substance i ($[n_{\text{i,R}}] = \text{mol}$) in the time between two points of measurement.

$$n_{\text{i,R}(t)} = \int_{t_{\text{j}-1}}^{t_{\text{j}}} \frac{\Delta\dot{n}_{\text{i},(t_{\text{j}})} - \Delta\dot{n}_{\text{i},(t_{\text{j}-1})}}{t_{\text{j}} - t_{\text{j}-1}} t + \left(\Delta\dot{n}_{\text{i},(t_{\text{j}-1})} - \frac{\Delta\dot{n}_{\text{i},(t_{\text{j}})} - \Delta\dot{n}_{\text{i},(t_{\text{j}-1})}}{t_{\text{j}} - t_{\text{j}-1}} t_{\text{j}-1}\right) dt$$

$$n_{\text{i,R}(t)} = \frac{\Delta\dot{n}_{\text{i},(t_{\text{j}})} - \Delta\dot{n}_{\text{i},(t_{\text{j}-1})}}{2(t_{\text{j}} - t_{\text{j}-1})} ({t_{\text{j}}}^2 - {t_{\text{j}-1}}^2) + \left(\Delta\dot{n}_{\text{i},(t_{\text{j}-1})} - \frac{\Delta\dot{n}_{\text{i},(t_{\text{j}})} - \Delta\dot{n}_{\text{i},(t_{\text{j}-1})}}{t_{\text{j}} - t_{\text{j}-1}} t_{\text{j}-1}\right)(t_{\text{j}} - t_{\text{j}-1}) + C$$

For $t_{\text{j}} \rightarrow t_{\text{j-1}}$: $\lim_{t_{\text{j}} \to t_{\text{j}-1}} n_{\text{i,R}(t)} = n_{\text{i,R}(t_{\text{j}-1})} \Rightarrow C = n_{\text{i,R}(t_{\text{j}-1})}$

$$n_{\text{i,R}(t)} = \frac{\Delta\dot{n}_{\text{i},(t_{\text{j}})} - \Delta\dot{n}_{\text{i},(t_{\text{j}-1})}}{2(t_{\text{j}} - t_{\text{j}-1})} ({t_{\text{j}}}^2 - {t_{\text{j}-1}}^2) + \left(\Delta\dot{n}_{\text{i},(t_{\text{j}-1})} - \frac{\Delta\dot{n}_{\text{i},(t_{\text{j}})} - \Delta\dot{n}_{\text{i},(t_{\text{j}-1})}}{t_{\text{j}} - t_{\text{j}-1}} t_{\text{j}-1}\right)(t_{\text{j}} - t_{\text{j}-1}) + n_{\text{i,R}(t_{\text{j}-1})}$$

Using the third binomial formula $t_{\text{j}}^2 - t_{\text{j-1}}^2 = (t_{\text{j}} + t_{\text{j-1}})(t_{\text{j}} - t_{\text{j-1}})$ the final equation is

$$n_{\text{i,R}(t)} = n_{\text{j,R}(t_{\text{j}-1})} + \frac{\Delta\dot{n}_{\text{i},(t_{\text{j}})} - \Delta\dot{n}_{\text{i},(t_{\text{j}-1})}}{2}(t_{\text{j}} + t_{\text{j}-1}) + \left(\Delta\dot{n}_{\text{i},(t_{\text{j}-1})} - \frac{\Delta\dot{n}_{\text{i},(t_{\text{j}})} - \Delta\dot{n}_{\text{i},(t_{\text{j}-1})}}{t_{\text{j}} - t_{\text{j}-1}} t_{\text{j}-1}\right)(t_{\text{j}} - t_{\text{j}-1}).$$
